# Differential Effects of Five Rearing Systems on Immune-Related Gene Expression in the Blood and Spleen of Termond White Rabbits

**DOI:** 10.3390/genes17040451

**Published:** 2026-04-13

**Authors:** Zuzanna Siudak, Paweł Bielański, Katarzyna Ropka-Molik, Katarzyna Piórkowska, Dorota Kowalska

**Affiliations:** 1 Department of Small Livestock Breeding, National Research Institute of Animal Production, 32-083 Balice, Poland; pawel.bielanski@iz.edu.pl (P.B.); dorota.kowalska@iz.edu.pl (D.K.); 2 Department of Animal Molecular Biology, National Research Institute of Animal Production, 32-083 Balice, Poland; katarzyna.piorkowska@iz.edu.pl

**Keywords:** Termond White rabbits, housing system, welfare, immune response, cytokines, gene expression, stress

## Abstract

Background/Objectives: Improving rabbit welfare through alternative housing systems requires a better understanding of how environmental conditions modulate physiological and immune responses at the molecular level. This study aimed to evaluate the influence of different rearing systems on the expression of genes associated with inflammation, immune regulation, and stress response in Termond White rabbits. Methods: After weaning (35 days of age), Termond White females (*n* = 16 per group) were allocated to five housing systems differing in space allowance and activity opportunities: hutches with outdoor runs, rabbit tractor cages with outdoor runs, single-floor indoor cages without bedding, indoor pens on deep litter, and modified indoor cages (two cages connected with a plastic pipe). At slaughter weight (2600–2900 g; 90–120 days), blood and spleen samples were collected. The relative expression of *IL6*, *CXCR1*, *IL10*, *TGFB1*, *IL8*, *PTGS2*, *IL1B*, and *TNF* was quantified by RT-qPCR using the 2^−ΔΔCt^ method, with ACTB and B2M as reference genes. Results: The housing system significantly affected the expression of most analysed genes in peripheral blood (*IL6*, *CXCR1*, *IL1B*, *PTGS2*, *IL8*, *TNF*, and *IL10*; *p* ≤ 0.05), whereas in the spleen significant differences were observed only for selected genes (*IL1B*, *TNF*, *CXCR1*, *IL10*, and *TGFB1*), with no effect detected for *IL6*, *IL8*, and *PTGS2* (*p* > 0.05). In blood, system-dependent differences were observed for both pro-inflammatory and regulatory genes, with some housing conditions associated with higher expression of inflammatory markers. In the spleen, the response was more selective and gene-specific, suggesting tissue-dependent modulation of immune-related pathways. Conclusions: Rearing environment influences the expression of immune-related genes in Termond White rabbits; however, these effects appear to be tissue-dependent and vary among specific genes. The observed transcriptional changes suggest potential associations between housing conditions and immune responses, but further studies integrating behavioural, physiological, and protein-level data are required to confirm their relevance for animal welfare assessment.

## 1. Introduction

In recent decades, rabbit production systems have undergone substantial changes driven by increasing consumer expectations and evolving welfare standards [1,2]. While consumption continues to rise in some regions, several European countries with established rabbit production systems have reported declines in production, accompanied by the introduction of alternative housing systems aimed at improving animal welfare. These systems differ markedly in structural design, space allowance, environmental complexity and exposure to outdoor conditions, all of which may influence physiological and behavioural responses in rabbits [3,4]. Housing conditions have been shown to affect growth performance, carcass traits and meat quality in rabbits [1,5]. At the same time, increasing attention has been given to animal welfare, as housing systems determine not only productivity but also animals’ comfort, behavioural expression and exposure to environmental stressors [1,4,6]. Ensuring optimal housing conditions is therefore essential not only from an ethical perspective but also for maintaining animal health, resilience and long-term physiological stability [6].

Although the effects of housing systems on production traits and welfare indicators have been widely studied, their impact on molecular and transcriptional mechanisms in rabbits remains poorly understood. In particular, there is limited information on how different housing environments modulate the expression of immune-related genes, which play a key role in the regulation of inflammatory and stress responses [7,8,9]. Moreover, most available studies focus on a limited number of housing systems or single tissues, leaving the tissue-specific nature of transcriptional responses largely unexplored.

The interaction between genotype and environment is increasingly recognised as a determinant of adaptive capacity, influencing transcriptional regulation, immune competence and long-term physiological stability [10]. External stimuli, including differences in spatial complexity, locomotor activity, microclimate and social environment, may induce transcriptional reprogramming of genes involved in immune regulation and stress response [9]. Such environmentally induced modulation of transcriptional activity enables organisms to maintain physiological homeostasis and to adjust immune function in response to environmental constraints [11]. Repeated or persistent environmental challenges may further reshape immune network organisation, leading to stable alterations in gene expression profiles that reflect adaptive or maladaptive responses [12].

In intensive and semi-intensive rabbit production systems, housing conditions differ markedly in structural design, stocking density, flooring type and exposure to outdoor factors [13]. These environmental variables may function as chronic or intermittent stimuli influencing systemic immune activation and regulatory mechanisms [14]. Although the impact of housing systems on productive traits has been extensively documented [1,15,16,17], their effects on animal welfare and physiological responses have also been recognised [6,18]. However, considerably less attention has been devoted to the transcriptional mechanisms underlying environmental adaptation in rabbits. Characterising environmentally responsive gene networks is essential for understanding the molecular basis of adaptive capacity and immune resilience [19]. Cytokines and chemokine receptors form interconnected signalling networks that coordinate innate and adaptive immune responses [20]. Pro-inflammatory cytokines, including interleukin 6 (IL6), interleukin 1 beta (IL1B), tumour necrosis factor (TNF) and interleukin 8 (IL8), are central mediators of inflammatory signalling cascades and are frequently regulated through NF-κB- and MAPK-dependent pathways [21,22]. Prostaglandin-endoperoxide synthase 2 (PTGS2) contributes to inflammation-associated prostaglandin synthesis and may amplify local and systemic immune responses [23]. Chemokine receptor CXCR1 plays a critical role in neutrophil recruitment and chemotactic signalling [24]. In contrast, regulatory cytokines such as interleukin 10 (IL10) and transforming growth factor beta 1 (TGFB1) are key mediators of the resolution and negative regulation of inflammatory responses and contribute to the maintenance of immune homeostasis [25]. The balance between pro-inflammatory and regulatory gene expression reflects the functional state of immune signalling networks and may provide insight into the organism’s response to environmental conditions [8]. Importantly, gene expression responses may differ between tissues, reflecting compartment-specific regulation of systemic and lymphoid immune activity; however, such tissue-dependent responses to housing conditions have not been sufficiently investigated in rabbits [26].

The Termond White rabbit, commonly used in commercial production systems, represents an appropriate model for investigating environmentally induced transcriptional modulation of immune-related genes [27]. This breed has been selected primarily for growth performance and carcass traits, and long-term directional selection may have shaped metabolic and physiological characteristics influencing immune responsiveness [28]. Evaluating transcriptional responses in this genotype may therefore provide insight into how commercially selected animals regulate immune-related gene networks under varying environmental conditions [7].

It was hypothesised that variation in housing environment would result in tissue-specific modulation of pro-inflammatory and regulatory transcripts, reflecting differential activation of immune signalling pathways and demonstrating environmentally driven transcriptional plasticity. The objective of the present study was to investigate the effect of five distinct rearing systems on the expression of selected immune-related genes (*CXCR1*, *IL10*, *IL8*, *IL1B*, *TNF*, *PTGS2*, *TGFB1*, *IL6*) in peripheral blood and spleen of Termond White rabbits. By combining multiple housing systems, a panel of inflammatory and regulatory genes, and two functionally distinct tissues, this study provides an integrated assessment of environment-dependent transcriptional responses in rabbits.

## 2. Materials and Methods

### 2.1. Animals and Experimental Design

**Figure 1:**
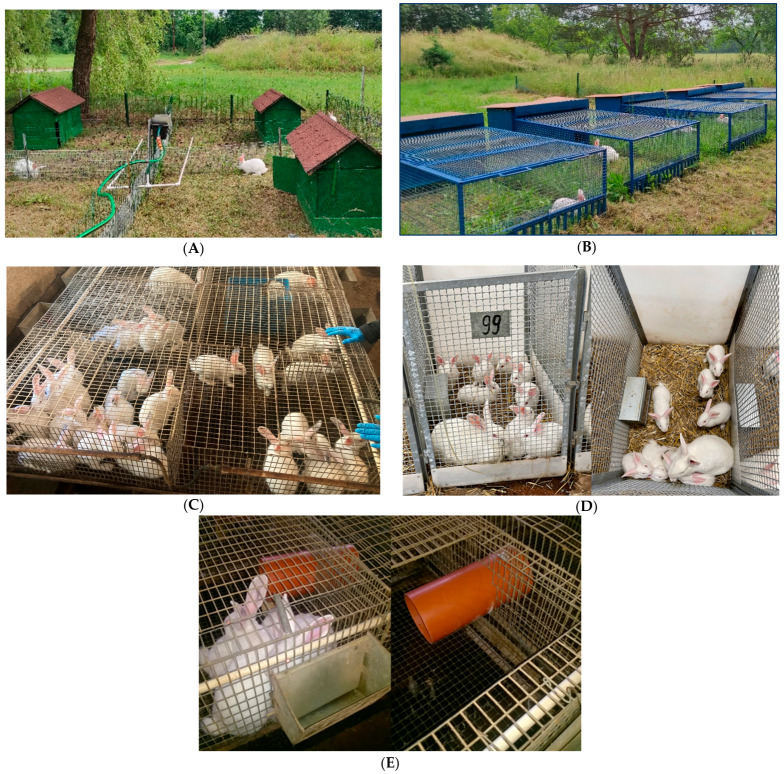
Rabbit housing systems. Rabbit hutches with an outdoor run at the Experimental Station of the National Research Institute of Animal Production, Chorzelów Ltd., Chorzelów, Poland (**A**); rabbit tractor cages at the Experimental Station of the National Research Institute of Animal Production, Chorzelów Ltd., Chorzelów, Poland (**B**); single-floor cages without litter at the Experimental Station of the National Research Institute of Animal Production, Chorzelów Ltd., Chorzelów, Poland (**C**); pens with deep litter at the National Research Institute of Animal Production in Aleksandrowice (**D**); modified cages—two cages connected with a plastic pipe at the Experimental Station of the National Research Institute of Animal Production, Chorzelów Ltd., Chorzelów, Poland (**E**).

The experiment was conducted at the National Research Institute of Animal Production in Aleksandrowice and at the Experimental Station of the National Research Institute of Animal Production in Chorzelów, Poland. The study was carried out from May to September. The experimental material consisted exclusively of Termond White (TW) female rabbits. At weaning (35 days of age), animals were allocated to five housing systems differing in structural design and environmental exposure. Each housing system comprised 16 females. Rabbits were housed in groups of four animals per cage or pen. Consequently, each housing system comprised four cages or pens, which were regarded as independent housing units. The housing systems were as follows: A—hutches with an outdoor run; B—rabbit tractor cages with an outdoor run; C—single-floor cages without bedding inside a livestock building; D—pens on deep litter inside a livestock building; E—modified cages inside a livestock building (two cages connected with a plastic pipe) (Figure 1). The mean body weight at weaning was 964 ± 112 g.

Standard cages measured 60 × 80 cm, whereas pens measured 120 × 80 cm. Outdoor enclosures measured 200 × 100 cm. Indoor rabbits were maintained under natural gravitational ventilation, with ambient temperatures ranging from 14 to 18 °C, relative humidity of 50–65%, and a photoperiod of 14 h light and 10 h dark (14L:10D). The mean outdoor temperature ranged from 14 to 22 °C. All housing conditions complied with Polish regulations concerning the keeping of farm and fur animals [29]. For gene expression analysis, eight rabbits per housing system were selected. Rabbits were chosen randomly, with two individuals sampled from each cage or pen to ensure representation across all housing units. Animals were included in a veterinary prophylaxis programme including vaccination against myxomatosis and rabbit haemorrhagic disease. No infectious disease outbreaks were recorded during the experimental period, and mortality did not exceed 3%.

### 2.2. Feeding and Slaughter Procedure

During the rearing period, rabbits were fed ad libitum with a complete pelleted diet. The chemical composition of the diet was analysed at the Central Laboratory of the National Research Institute of Animal Production. Digestible energy (DE) was calculated using the equation: DE (MJ/kg DM) = 0.016 × CP + 0.034 × EE + 0.014 × CF + 0.017 × NFE, where CP denotes crude protein, EE ether extract, CF crude fibre, and NFE nitrogen-free extract. Digestible protein (DP) was estimated as 0.75 × CP, and the DP/DE ratio (g/MJ) was calculated accordingly. The ingredient and chemical composition of the diet is presented in Table 1 and Table 2.

At a body weight of 2600–2900 g, selected animals were used for gene expression analysis as described above; this resulted in an age range of 90–120 days due to differences in growth rates between housing systems. Animals were fasted for 16 h with free access to water prior to slaughter. Slaughter procedures were carried out according to Blasco et al. [30]. Rabbits were humanely stunned before exsanguination. Peripheral whole blood samples were collected during bleeding from the severed carotid artery into Tempus™ Blood RNA Tubes (Thermo Fisher Scientific, Waltham, MA, USA). Spleen samples were collected immediately post mortem, snap-frozen in liquid nitrogen, and stored at −80 °C until further analysis. Blood samples were stored at −20 °C prior to RNA isolation.

### 2.3. Quantitative Real-Time PCR

Total RNA was isolated from blood samples using the PureLink™ RNA Mini Kit with the PureLink™ DNase Set (Invitrogen, Carlsbad, CA, USA). RNA samples were stored at –80 °C until analysis. RNA concentration and purity were assessed with a NanoDrop 2000 spectrophotometer (Thermo Scientific, Waltham, MA, USA), and integrity was verified by 2% agarose gel electrophoresis. cDNA was synthesised from 500 ng of total RNA using the High-Capacity RNA-to-cDNA Reverse Transcription Kit (Applied Biosystems, Foster City, CA, USA) according to the manufacturer’s protocol. Expression of *IL6*, *CXCR1*, *IL10*, *TGFB1*, *IL8*, *PTGS2*, *IL1B* and *TNF* was quantified using TaqMan assays (Applied Biosystems) on a QuantStudio 7 Flex Real-Time PCR System (Thermo Fisher Scientific, Waltham, MA, USA). Reactions were carried out in triplicate using the RT HS-PCR Mix Probe kit (A&A Biotechnology, Gdańsk, Poland) with *ACTB* and *B2M* as reference genes [31]. The TaqMan assays used were: *ACTB* (*Oc03824857_g1-ENDO*), *B2M (Oc06779339_m1-ENDO*), *CXCR1* (*Oc04250543_s1*), *IL10* (*Oc03396940_m1*), *IL8* (*Oc03397860_m1*), *IL1B* (*Oc03823250_s1*), *TNF* (*Oc03397715_m1*), *PTGS2* (*Oc03398295_m1*), *TGFB1* (*Oc04176122_u1*) and *IL6* (*Oc04097053_m1*). Thermal cycling conditions were 95 °C for 5 min, followed by 95 °C for 15 s and 60 °C for 45 s. Gene expression was analysed using the ΔΔCt method. Relative quantification (RQ) values were calculated as 2^−ΔΔCt^. Amplification efficiency for each assay was evaluated based on standard curves using the formula E = 10^(−1/slope) − 1 and confirmed to be within an acceptable range. Only assays with comparable amplification efficiencies were included in the analysis. Relative transcript abundance was normalised to reference genes and expressed relative to the calibrator sample with the lowest expression [32].

### 2.4. Statistical Analysis

Statistical analyses were performed in Statistica 13.1 (TIBCO Software Inc., Palo Alto, CA, USA). A mixed-effects model was applied, with housing system as a fixed effect and cage/hutch nested within housing system as a random effect. The statistical model was defined as: Yijk = μ + Hi + Cj(i) + eijk, where Yijk represents the observed gene expression value, μ is the overall mean, Hi is the effect of housing system, Cj(i) is the random effect of cage/hutch nested within housing system, and eijk is the random error. Statistical analyses were performed on relative expression values (RQ; 2^−ΔΔCt^). Although RQ values may deviate from normal distribution, the assumptions of normality (Shapiro–Wilk test) and homogeneity of variance (Levene’s test) were verified prior to analysis and did not indicate significant violations. Therefore, the data were considered suitable for parametric testing, and no additional transformations were applied. Additionally, exploratory analyses based on ΔCt values showed comparable patterns of results, supporting the robustness of the applied approach. Rabbits were treated as observational units nested within cages/hutches. Post hoc comparisons were conducted using pairwise comparisons between housing systems, with statistical significance set at *p* ≤ 0.05. Given that multiple genes and pairwise comparisons were analysed, no formal correction for multiple testing was applied; therefore, the results should be interpreted with caution due to the increased risk of type I error. Data are presented as means ± SD.

## 3. Results

### 3.1. Blood Gene Expression

**Figure 2:**
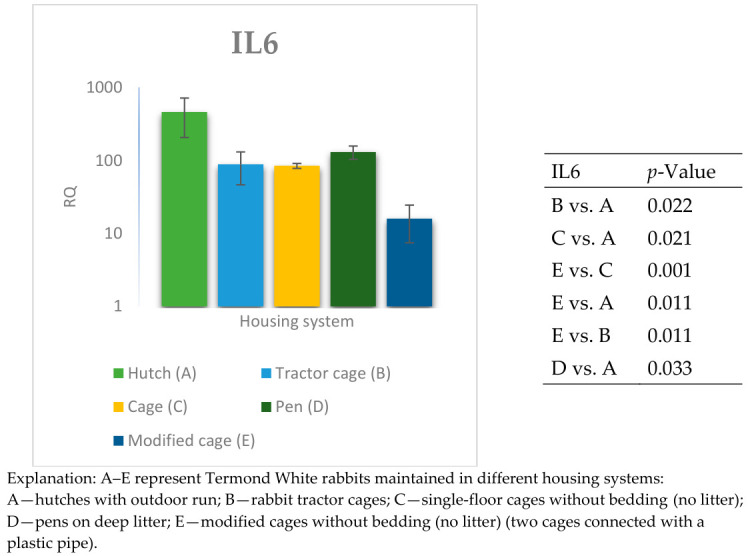
Effect of housing system on the relative expression of the *IL6* gene in the blood of Termond White (TW) rabbits (means ± SD) (RQ—Relative Quantity normalised to *ACTB* and *B2M*).

**Figure 3:**
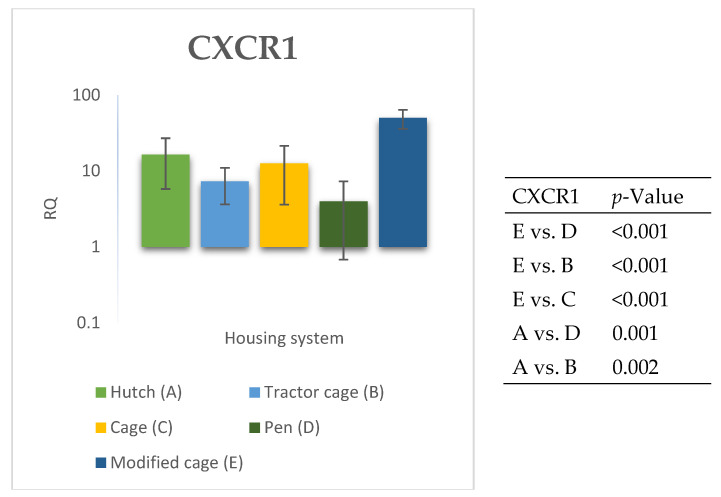
Effect of housing system on the relative expression of the *CXCR1* gene in the blood of Termond White (TW) rabbits (means ± SD) (RQ—Relative Quantity normalised to *ACTB* and *B2M*).

**Figure 4:**
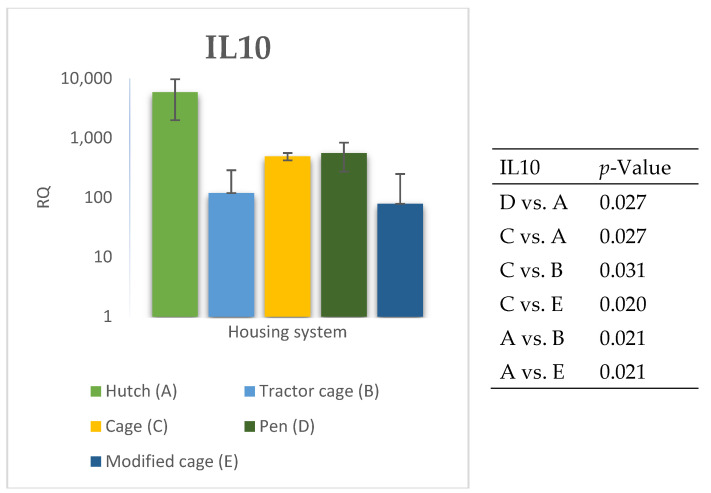
Effect of housing system on the relative expression of the *IL10* gene in the blood of Termond White (TW) rabbits (means ± SD) (RQ—Relative Quantity normalised to *ACTB* and *B2M*).

**Figure 5:**
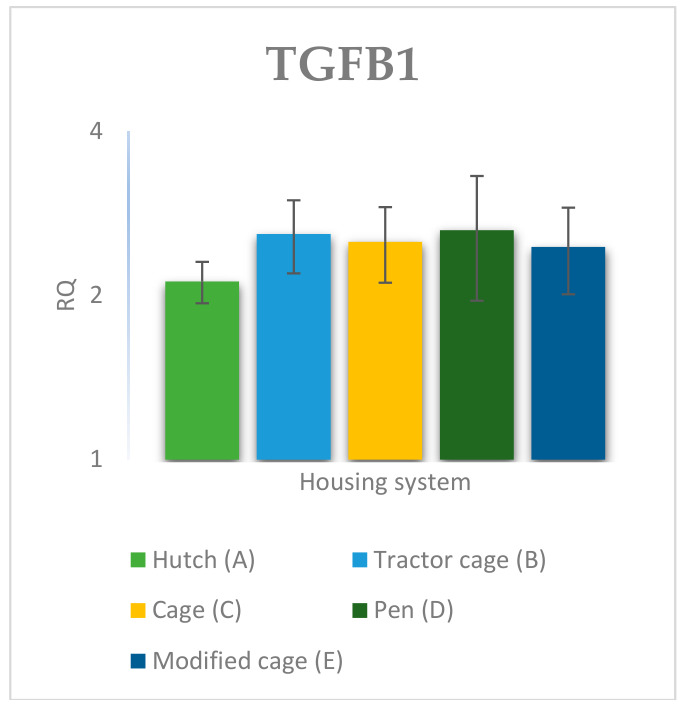
Effect of housing system on the relative expression of the *TGFB1* gene in the blood of Termond White (TW) rabbits (means ± SD) (RQ—Relative Quantity normalised to *ACTB* and *B2M*).

**Figure 6:**
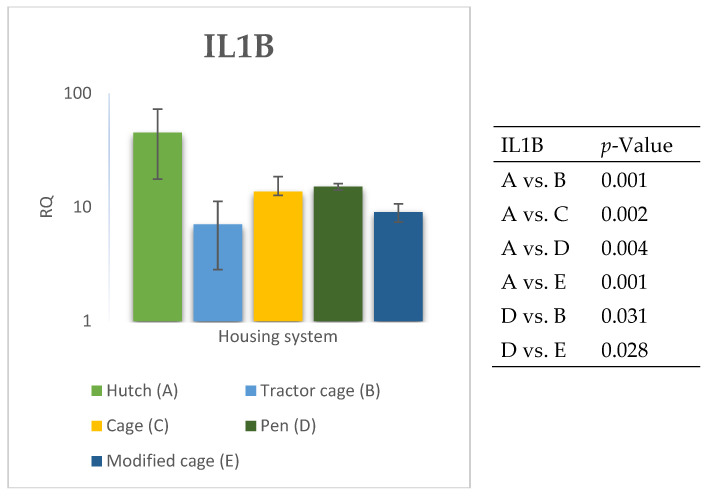
Effect of housing system on the relative expression of the IL1B gene in the blood of Termond White (TW) rabbits (means ± SD) (RQ—Relative Quantity normalised to *ACTB* and *B2M*).

**Figure 7:**
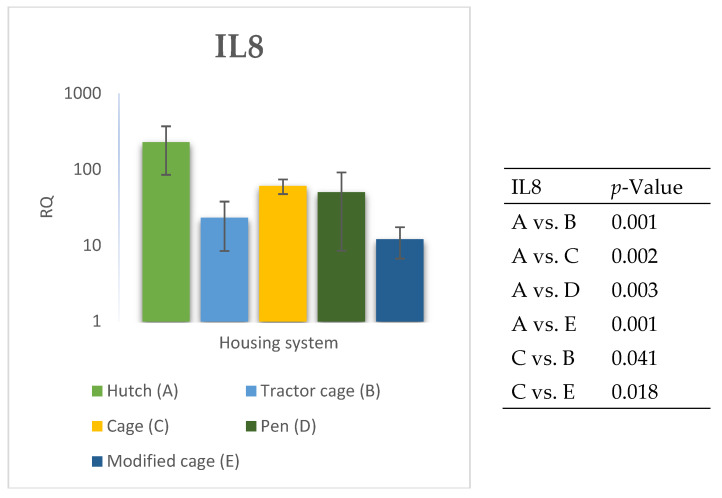
Effect of housing system on the relative expression of the *IL8* gene in the blood of Termond White (TW) rabbits (means ± SD) (RQ—Relative Quantity normalised to *ACTB* and *B2M*).

**Figure 8:**
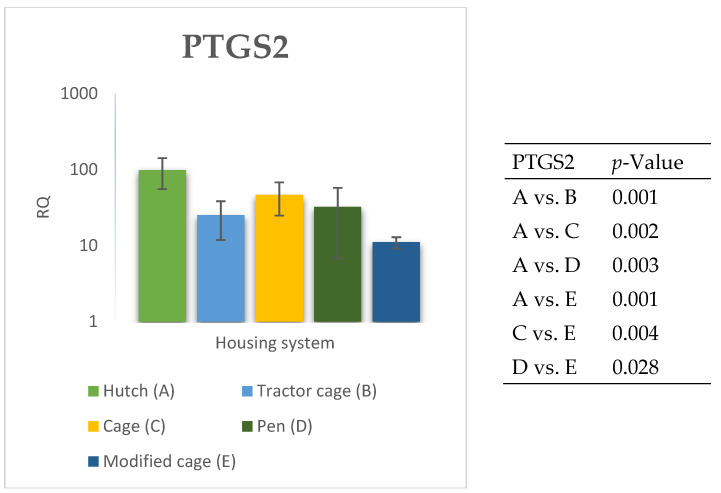
Effect of housing system on the relative expression of the *PTGS2* gene in the blood of Termond White (TW) rabbits (means ± SD) (RQ—Relative Quantity normalised to *ACTB* and *B2M*).

**Figure 9:**
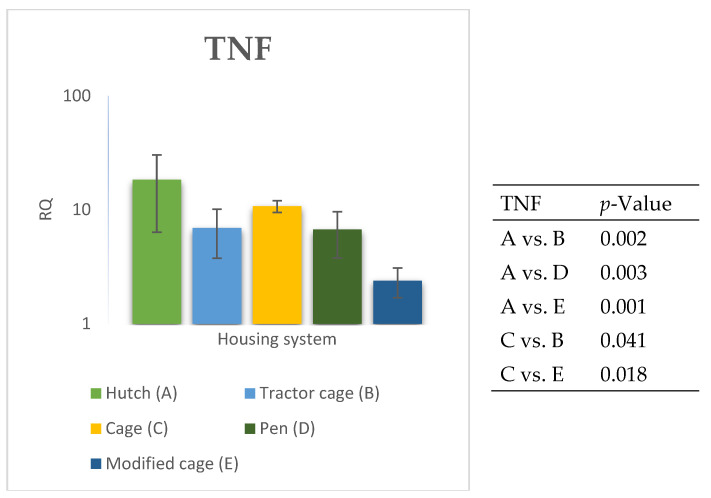
Effect of housing system on the relative expression of the *TNF* gene in the blood of Termond White (TW) rabbits (means ± SD) (RQ—Relative Quantity normalised to *ACTB* and *B2M*).

The relative expression levels of selected genes were analysed in the peripheral blood of Termond White rabbits maintained under different housing systems (A–E). The results are presented in Figure 2, Figure 3, Figure 4, Figure 5, Figure 6, Figure 7, Figure 8 and Figure 9. A significant effect of housing system was observed for *IL6*, *CXCR1*, *IL1B*, *PTGS2*, *IL8*, *TNF*, and *IL10* (*p* ≤ 0.05), whereas no significant differences were found for *TGFB1* (*p* > 0.05). Overall, the expression patterns revealed a strong effect of housing system on pro-inflammatory gene activation in blood. System A was generally associated with the highest expression levels of most pro-inflammatory genes (*IL6*, *IL1B*, *TNF*, *IL8*, and *PTGS2*), whereas other systems showed lower or intermediate values. *IL6* expression varied among housing systems, with system A showing higher values than all other systems (*p* ≤ 0.05). A similar pattern was observed for *IL1B*, where system A differed from the remaining systems and system D showed intermediate values (*p* ≤ 0.05). TNF transcript levels also differed between systems, with higher expression in system A compared to systems B, D, and E, while system C showed intermediate values (*p* ≤ 0.05). IL8 followed a comparable trend, with the highest levels observed in system A and intermediate expression in system C (*p* ≤ 0.05). For *PTGS2*, differences between housing systems were detected (*p* ≤ 0.05), with system A showing higher expression than the remaining systems, and systems C and D differing from system E. *CXCR1* expression showed a distinct pattern, with system E exhibiting the highest levels compared to all other systems, while system A showed higher values than systems B, C, and D (*p* ≤ 0.05). *IL10* expression also varied between housing systems, with system A showing higher values than the remaining systems, and system C differing from systems B and E (*p* ≤ 0.05).

### 3.2. Spleen Gene Expression

**Figure 10:**
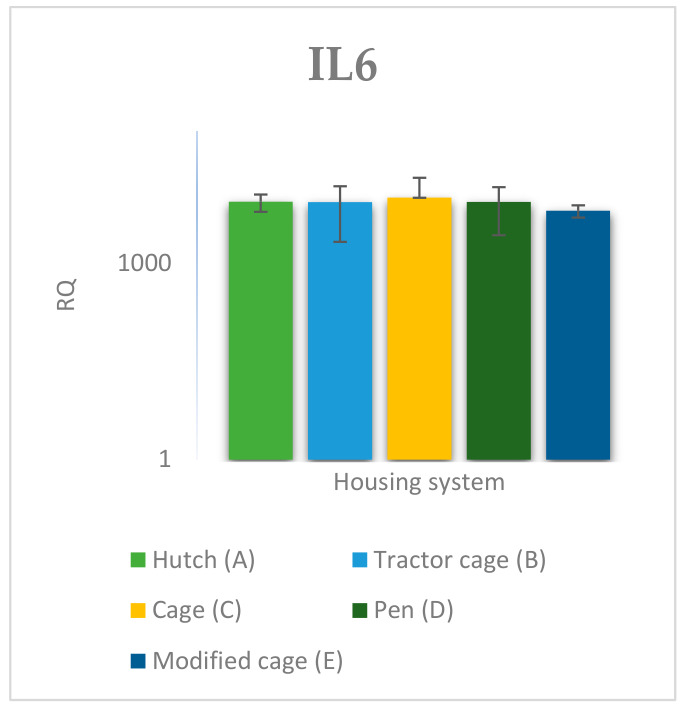
Effect of housing system on the relative expression of the *IL6* gene in the spleen of Termond White (TW) rabbits (means ± SD) (RQ—Relative Quantity normalised to *ACTB* and *B2M*).

**Figure 11:**
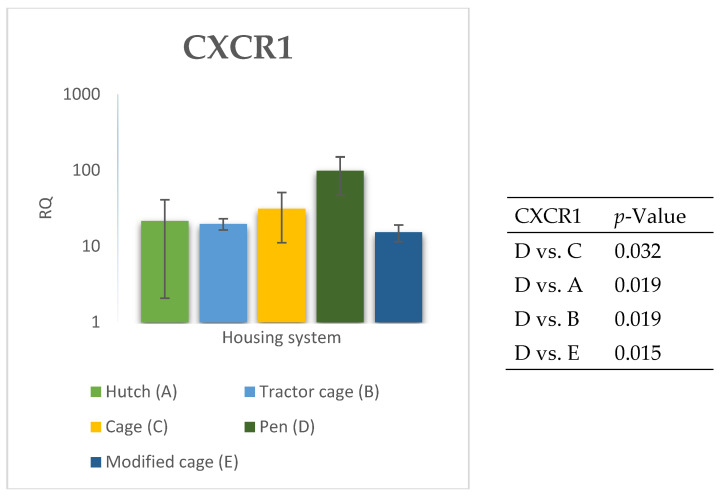
Effect of housing system on the relative expression of the *CXCR1* gene in the spleen of Termond White (TW) rabbits (means ± SD) (RQ—Relative Quantity normalised to *ACTB* and *B2M*).

**Figure 12:**
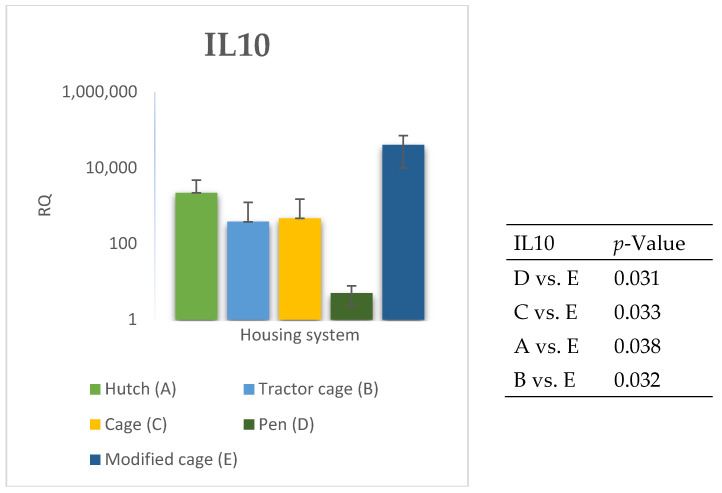
Effect of housing system on the relative expression of the *IL10* gene in the spleen of Termond White (TW) rabbits (means ± SD) (RQ—Relative Quantity normalised to *ACTB* and *B2M*).

**Figure 13:**
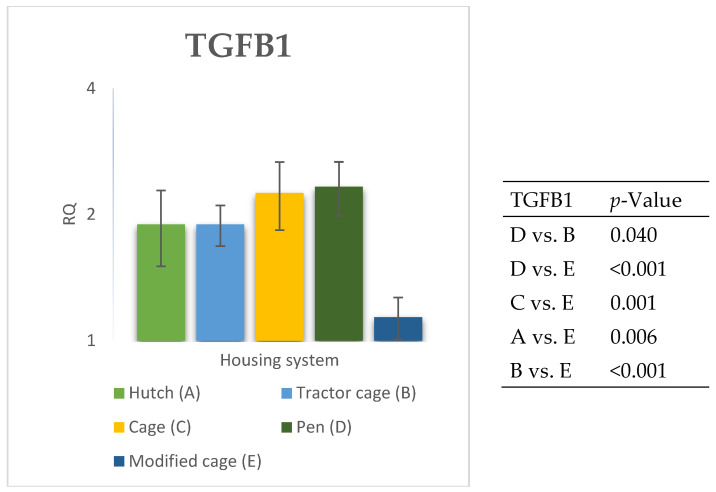
Effect of housing system on the relative expression of the *TGFB1* gene in the spleen of Termond White (TW) rabbits (means ± SD) (RQ—Relative Quantity normalised to *ACTB* and *B2M*).

**Figure 14:**
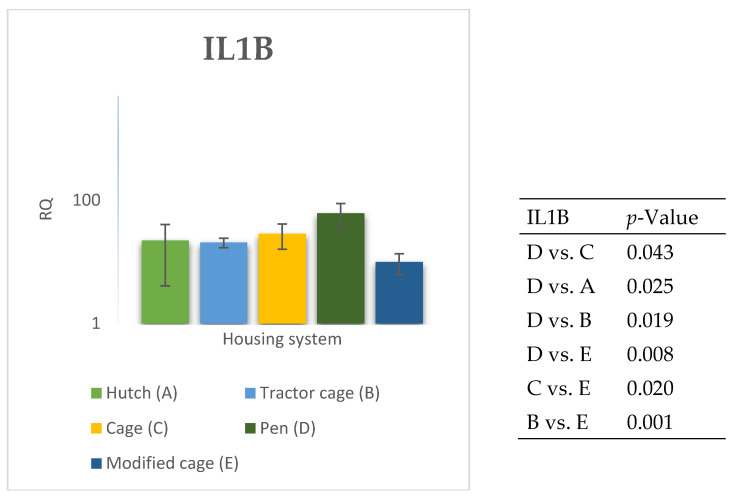
Effect of housing system on the relative expression of the *IL1B* gene in the spleen of Termond White (TW) rabbits (means ± SD) (RQ—Relative Quantity normalised to *ACTB* and *B2M*).

**Figure 15:**
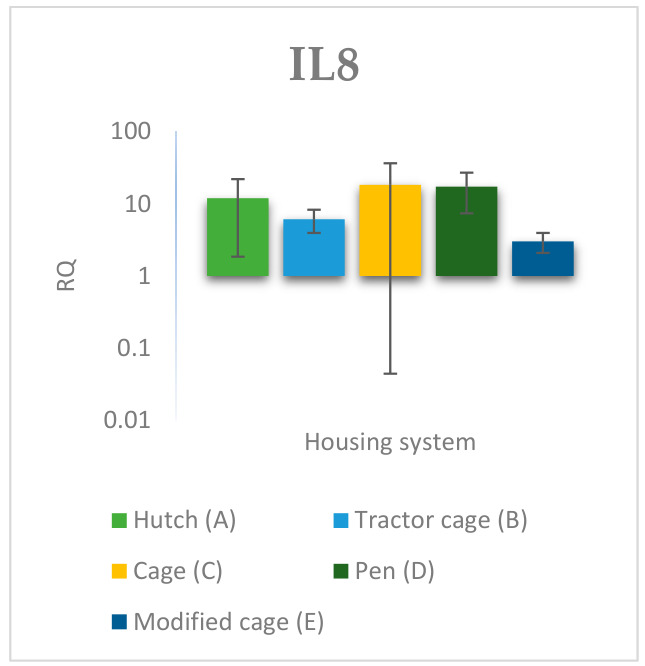
Effect of housing system on the relative expression of the *IL8* gene in the spleen of Termond White (TW) rabbits (means ± SD) (RQ—Relative Quantity normalised to *ACTB* and *B2M*).

**Figure 16:**
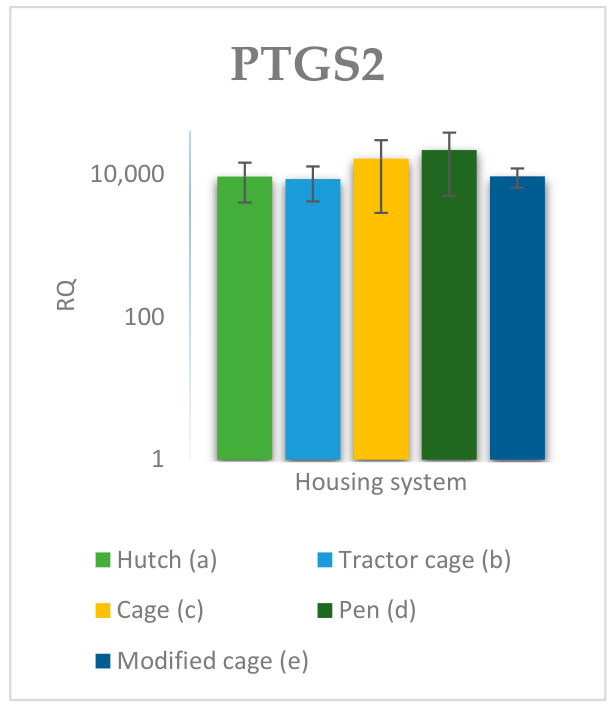
Effect of housing system on the relative expression of the *PTGS2* gene in the spleen of Termond White (TW) rabbits (means ± SD) (RQ—Relative Quantity normalised to *ACTB* and *B2M*).

**Figure 17:**
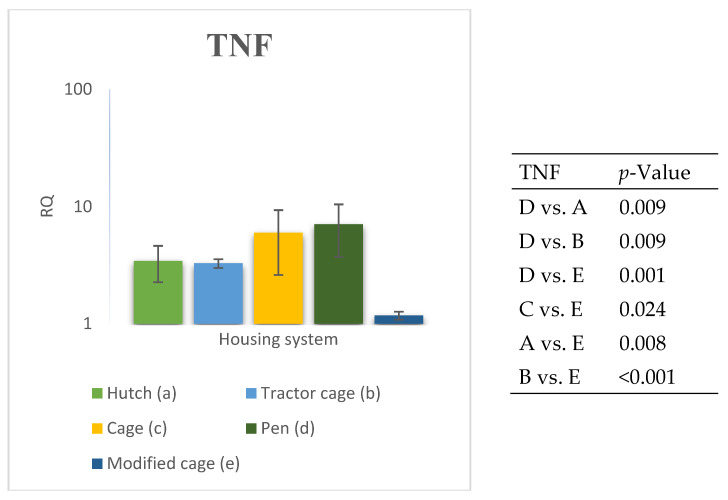
Effect of housing system on the relative expression of the *TNF* gene in the spleen of Termond White (TW) rabbits (means ± SD) (RQ—Relative Quantity normalised to *ACTB* and *B2M*).

The relative expression levels of selected genes were analysed in the spleen of Termond White rabbits maintained under different housing systems (A–E). The results are presented in Figure 10, Figure 11, Figure 12, Figure 13, Figure 14, Figure 15, Figure 16 and Figure 17. A significant effect of housing system was observed for *IL1B*, *TNF*, *CXCR1*, *IL10*, and *TGFB1* (*p* ≤ 0.05), whereas no significant differences were found for *IL6*, *IL8*, and *PTGS2* (*p* > 0.05). In contrast to blood, gene expression patterns in the spleen were more selective and gene-specific, with fewer genes affected by housing conditions. *TGFB1* expression varied among housing systems, with system E showing higher values than systems A–D, and system D differing from system B (*p* ≤ 0.05). *IL1B* showed clear variation, with system D exhibiting the highest levels compared to the other systems, while systems B and C differed from system E (*p* ≤ 0.05). *TNF* expression also differed between housing systems, with system D showing higher levels than systems A, B, and E, whereas system E exhibited the lowest values (*p* ≤ 0.05). *CXCR1* expression was consistently higher in system D compared to all other systems (*p* ≤ 0.05). IL10 expression varied between housing systems, with system E showing the highest values compared to the remaining systems (*p* ≤ 0.05). Overall, the spleen results indicate a more localised and selective modulation of immune-related genes, with system D associated with increased expression of inflammatory markers and system E with higher expression of regulatory genes.

### 3.3. Summary of Gene Expression Patterns Across Housing Systems

The summary presented in Table 3 highlights clear tissue-dependent patterns. In peripheral blood, system A was generally associated with higher expression of pro-inflammatory genes, whereas in the spleen, system D showed increased expression of inflammatory markers and system E was associated with higher expression of regulatory genes. Overall, gene expression responses were more pronounced in blood and more selective in the spleen.

## 4. Discussion

The study suggests that the rabbit housing system may influence the expression of genes associated with the inflammatory response and immune regulation, both in blood and in the spleen. The results obtained indicate that diverse environmental conditions give rise to divergent physiological responses, which may be indicative of varying levels of environmental stress, behavioural activity and adaptive capacity in animals [33]. Of particular significance was the high variability observed across groups, suggesting that the housing microenvironment may modulate the immune response of rabbits at multiple levels. A significant divergence in the expression of pro-inflammatory and migration-related genes was observed between the housing systems in peripheral blood, whereas in the spleen the response was limited to a smaller subset of genes. This pattern suggests that circulating immune responses may be more sensitive to environmental variation than those observed in lymphoid tissues [34]. The elevated expression of *IL6* in blood may indicate increased activation of inflammatory pathways under specific housing conditions, while no significant differences were observed for this gene in the spleen. *IL6* plays a pivotal role in the acute-phase response and frequently increases under conditions of environmental stress, which may reflect altered physiological responses to environmental stimuli [35]. Similar trends observed for *IL8* and *TNF* further support enhanced pro-inflammatory signalling in blood, potentially associated with neutrophil activation [36,37]. In blood, *IL1B* expression was strongly influenced by housing system, whereas in the spleen a more pronounced response observed under specific housing conditions suggests tissue-specific activation of innate immune processes. The observed differences between housing systems may reflect modulation of inflammatory signalling pathways, potentially related to behavioural stress or microclimate conditions [38].

The high expression of *PTGS2* observed in several housing systems may reflect elevated inflammatory and stress processes [39]. *PTGS2* is responsible for synthesising prostaglandins, which play a key role in directing immune cells to the site of inflammation. However, while *PTGS2* expression differed significantly between housing systems in blood, no significant differences were detected in the spleen, suggesting that prostaglandin-mediated responses may be more pronounced in circulating cells than in lymphoid tissues. The differences in expression observed between housing systems further emphasise that the inflammatory response may vary depending on the maintenance conditions, which may have implications for animal health [40]. Differences were also observed in the expression of regulatory genes, suggesting variation in immunoregulatory activity between housing conditions. *IL10*, the primary inhibitor of the inflammatory response, exhibited higher expression in some housing systems, suggesting the activation of mechanisms balancing inflammatory processes in animals exposed to stronger environmental stimuli [7]. In particular, *IL10* expression observed in the spleen suggests enhanced anti-inflammatory regulation in this tissue. *TGFB1*, a key regulator of immune cell differentiation and proliferation, showed variable expression between housing systems, which may indicate differences in the intensity of adaptive processes in animals maintained under different environmental conditions [41]. Unlike in blood, where no significant differences were observed, *TGFB1* expression in the spleen differed significantly between systems, highlighting tissue-specific regulation of immune responses.

Differences between housing systems were also observed in the spleen gene expression profiles, the main lymphatic organ responsible for the adaptive response [42]. In contrast to blood, not all analysed genes responded to housing conditions in the spleen, indicating a more selective and regulated transcriptional response in this tissue. *TGFB1* expression varied between housing systems, suggesting differences in immunoregulatory activity in response to environmental stimuli [43]. *IL1B* expression may reflect activation of innate immune mechanisms in some housing conditions, whereas lower expression in other systems suggested reduced inflammatory activation. Similarly, *TNF* expression varied between housing conditions, whereas *IL8* expression remained unchanged in the spleen despite differences observed in blood. This pattern further supports the notion that environmental factors may differentially modulate immune responses at the level of lymphoid tissues.

Overall, the expression patterns observed across both tissues, as summarised in Table 3, indicate that different housing systems may influence the balance between pro-inflammatory and regulatory immune mechanisms. Some systems were associated with stronger activation of pro-inflammatory pathways (*IL6*, *IL1B*, *TNF*, *IL8* and *PTGS2*), whereas others were characterised by relatively higher expression of regulatory genes such as *IL10* and *TGFB1*. Importantly, these effects were more pronounced and widespread in blood, while in the spleen they were more selective and gene-specific, reflecting differences in the functional roles of these tissues. In general, housing conditions associated with increased expression of pro-inflammatory genes in blood may indicate enhanced systemic immune activation. In contrast, conditions characterised by higher expression of regulatory markers, particularly in the spleen, may reflect a shift towards anti-inflammatory regulation. Intermediate expression patterns observed in some systems suggest that the immune response to housing conditions is not uniform but depends on the specific environmental context.

Beloumi et al. [44] described similar relationships between the ability of animals to cope with environmental stress and immune system activation. They studied the inflammatory response in two lines of rabbits selected for extreme variability in litter size. The authors demonstrated that the line with greater environmental variability had higher concentrations of TNF-α and significantly higher levels of CRP, as well as lower leucocyte counts, compared to the line with more stable litter size. This indicates greater susceptibility to infection and a weaker ability to maintain immunological homeostasis under stressful conditions. These results emphasise that differences in the inflammatory response of rabbits may result from environmental conditions and genetic factors, as well as differences in innate sensitivity to stress. In agreement with these findings, our results indicate that housing systems associated with higher expression of pro-inflammatory genes in blood also showed signs of enhanced immune activation, whereas the spleen exhibited a more regulated response. Comparing these observations with our results suggests that differences in the immune reactivity of rabbits may result from a complex interaction between the rearing environment and genetic characteristics.

Trocino and Xiccato [4] highlight that the housing system is a key factor in modulating physiological stress indicators, behaviour, and susceptibility to disease in rabbits. They also emphasise that inappropriate environmental conditions can result in disorders of homeostasis, changes in the neuroendocrine system, and a weakened immune response. They point out that such conditions, including limited space, lack of opportunity to express natural behaviours, inadequate cage height and insufficient environmental stimulation, can result in chronic stress. This is associated with a weakened immune system and increased susceptibility to disease, for example through changes in leucocyte count. In the context of our results, this may explain why different housing systems induced different levels of expression of inflammatory genes, particularly *IL6*, *IL8*, *TNF* and *PTGS2*, which are known markers of stress and activation of the inflammatory response. Housing systems that provide better behavioural conditions and reduce environmental stress may be associated with reduced cytokine expression. In contrast, more restrictive conditions may promote increased immune system activation. Thus, our results suggest the importance of housing environment quality as a key factor influencing rabbit immunity and welfare.

Another factor that may contribute to the differences observed between housing systems is the microbiome–immune axis, which has recently been identified as a key regulator of inflammatory responses in rabbits and other monogastric herbivores. The composition and stability of the gut microbiota are highly sensitive to environmental conditions, stress, diet and animal behaviour, all of which can differ between housing systems [45]. Changes in the structure of the microbial population are strongly correlated with the expression of pro-inflammatory cytokines such as IL6, TNF and IL1B, as well as regulatory mediators including IL10 and TGFB1 [46]. Dysbiosis, which is often triggered by environmental stressors or limited behavioural opportunities, can increase intestinal permeability and promote systemic immune activation [42]. Conversely, a stable and diverse microbiota is associated with lower inflammatory tone and more efficient immunoregulation. Wu et al. [47] demonstrated that different rearing systems markedly shape the gut microbiome, metabolome and intestinal transcriptome of rabbits. Complex environments were found to promote stronger activation of immune-related genes and distinct microbial signatures linked to immune modulation. Additionally, the study revealed positive correlations between specific bacterial taxa, such as *Erysipelotrichaceae* and *Delftia*, and immune-related transcripts. This finding suggests that the composition of the microbiota can either amplify or buffer host immune responses depending on environmental conditions.

Despite the observation of numerous significant differences between housing systems in both tissues, the expression patterns were not always identical. This is consistent with the different roles of these tissues in the immune response: while blood reflects dynamic and short-term processes, the spleen represents a more stable response associated with lymphocyte activation and differentiation [42,48].

Neves et al. [49] carried out a thorough genetic analysis of ten interleukins in a variety of lagomorph species. They demonstrated that cytokines such as IL-1α, IL-1β, IL-8 and IL-10 are highly conserved within the *Oryctolagus* genus, whereas sequence and structural variations occur between species and could impact immune function. The study emphasises that rabbits exhibit considerable diversity in the genes responsible for regulating inflammatory responses, and that even minor alterations, such as changes in glycosylation motifs or cysteine numbers, can affect the efficacy of immune responses and the activation of pro- and anti-inflammatory pathways. These findings support the interpretation that differences in inflammatory gene expression may result from both environmental conditions and intrinsic genetic regulation of immune pathways.

The results obtained suggest that housing conditions may influence immune-related gene expression. Groups with high expression of inflammatory cytokines may be associated with an increased risk of low-grade inflammation, whereas systems characterised by higher expression of regulatory genes (e.g., *TGFB1*, *IL10*) may be associated with a more balanced immune profile. However, these effects appear to be tissue-dependent, with more consistent and pronounced responses observed in blood compared to the spleen. These results highlight the potential importance of optimising environmental conditions in rabbit husbandry to improve welfare and maintain proper immune system function. Several limitations of the present study should be acknowledged. The analysis only considers gene expression levels, which do not necessarily correspond directly to protein levels. Therefore, future studies should determine cytokine levels in plasma or tissue homogenates. In addition, although *ACTB* and *B2M* were selected as reference genes based on previous studies, their stability was not formally validated under the specific experimental conditions of this study, which may influence the accuracy of normalisation. Furthermore, given that multiple genes and pairwise comparisons were analysed without formal correction for multiple testing, the results should be interpreted with caution due to the increased risk of type I error. Detailed data on behaviour, microclimate and oxidative stress are also required in order to explain the mechanisms underlying the observed differences. Further studies could also consider changes in the cellular composition of the blood and spleen, as well as the dynamics of the immune response in subsequent weeks of rearing.

## 5. Conclusions

This study suggests that the housing system markedly modulates the expression of inflammatory and regulatory genes in the blood and spleen of Termond White rabbits. The effects of housing system were more pronounced in peripheral blood, where significant differences were observed for most analysed genes, whereas in the spleen the response was more selective and limited to specific genes. System A was associated with strong activation of pro-inflammatory genes in blood (*IL6*, *IL1B*, *TNF*, *IL8* and *PTGS2*), which may reflect increased systemic immune activation. System D showed elevated expression of selected inflammatory genes, particularly in the spleen (e.g., *IL1B*, *TNF* and *CXCR1*), suggesting enhanced local immune responses. In contrast, system E was characterised by higher expression of regulatory genes such as *IL10*, especially in the spleen, which may indicate a shift towards anti-inflammatory regulation. Systems B and C generally showed intermediate expression profiles. These findings indicate that housing conditions differentially modulate systemic and organ-specific immune responses, and that the balance between pro-inflammatory and regulatory pathways may be associated with the type of housing system. Gene expression profiling of peripheral blood and spleen may represent a sensitive tool for detecting subclinical effects of housing on immune function and may complement traditional welfare and performance indicators in rabbit husbandry. The results highlight the potential importance of considering environmental conditions when designing welfare-oriented housing systems for Termond White rabbits. Future studies should link gene expression with cytokine protein levels, microbiome characteristics and detailed behavioural observations to refine molecular indicators of welfare and to optimise rearing conditions for this breed.

## Figures and Tables

**Table 1:** Component composition of feed mixture fed to rabbits.

Component	%
Post-extraction soybean meal	13.00
Lucerne meal	20.10
Wheat bran	18.10
Ground barley	28.80
Ground maize	11.00
Rapeseed oil	2.00
Phosphate	1.00
NaCl	0.50
Mineral and vitamin premix	1.00
Arbocel	4.50

**Table 2:** Chemical composition of feed mixture.

Chemical Composition	Content
Dry matter (%)	90.64
Crude ash (%)	6.92
Crude fat (%)	4.19
Crude protein (%)	15.20
Crude fibre (%)	14.2
aNDF (%)	25.6
ADF (%)	14.4
ADL (%)	2.95
NFE (%)	50.13
DE (MJ/kg DM)	13.45
DP/DE (g/MJ)	0.85

Explanation: NDF—neutral detergent fibre; ADF—acid detergent fibre; ADL—acid detergent lignin; NFE—nitrogen-free extract; DE—digestible energy, DP—digestible protein.

**Table 3:** Summary of gene expression patterns across housing systems (A–E) in peripheral blood and spleen of Termond White rabbits. “ns” indicates no significant differences between housing systems.

Gene	Blood—Highest Expression	Spleen—Highest Expression	General Pattern
*IL6*	A	ns	Blood-specific increase
*IL1B*	A	D	System A (blood), D (spleen)
*TNF*	A	D	Consistent inflammatory response
*IL8*	A	ns	Blood-specific response
*PTGS2*	A	ns	Blood-specific response
*CXCR1*	E	D	Tissue-dependent
*IL10*	A	E	Regulatory shift in spleen
*TGFB1*	ns	E	Spleen-specific regulation

Explanation: The table includes only housing systems with the highest expression values for each gene. A–E represent Termond White rabbits maintained in different housing systems: A—hutches with outdoor run; B—rabbit tractor cages; C—single-floor cages without bedding (no litter); D—pens on deep litter; E—modified cages without bedding (no litter) (two cages connected with a plastic pipe).

## Data Availability

The raw data are available from the corresponding authors upon reasonable request.
